# Low-volume goat milk transmission of classical scrapie to lambs and goat kids

**DOI:** 10.1371/journal.pone.0204281

**Published:** 2018-09-20

**Authors:** Sally A. Madsen-Bouterse, Margaret A. Highland, Rohana P. Dassanayake, Dongyue Zhuang, David A. Schneider

**Affiliations:** 1 Department of Veterinary Microbiology and Pathology, College of Veterinary Medicine, Washington State University, Pullman, Washington, United States of America; 2 Animal Disease Research Unit, Agricultural Research Service, United States Department of Agriculture, Pullman, Washington, United States of America; 3 Washington Animal Disease Diagnostic Laboratory, College of Veterinary Medicine, Washington State University, Pullman, Washington, United States of America; 4 Paul G. Allen School for Global Animal Health, College of Veterinary Medicine, Washington State University, Pullman, Washington, United States of America; Deutsches Zentrum fur Neurodegenerative Erkrankungen, GERMANY

## Abstract

The risk of classical scrapie transmission in small ruminants is highest during the neonatal period with the placenta recognized as a significant source of infection. Milk has also been identified as a source of scrapie with sheep-to-sheep transmission occurring after neonatal consumption of as little as 1–2 liters of milk; concurrent mastitis due to small ruminant lentivirus (SRLV) infection may be associated with increased scrapie transmission via milk in sheep. In contrast, goat-to-sheep transmission has been documented only after prolonged consumption of >30 liters of milk. The goal of the current study was to assess transmission of scrapie to goat kids and lambs following low volume, short duration consumption of milk from infected goats. Milk from two does (female goats) with pre-clinical scrapie was fed to four goat kids (≤4.5 L each) and four lambs (~3.7 L each) beginning ~24 hours after birth. Scrapie transmission was detected in three sheep as early as 18 months post inoculation; transmission was also detected in two goats but not until postmortem analyses at 33 months post inoculation. Each milk donor goat also had naturally-acquired infection with SRLV. Different degrees of lymphohistiocytic inflammation and PrP^Sc^ accumulation were observed in mammary gland tissues of the donors, which appeared to associate with transmission of scrapie via milk. Thus, similar to the risks of milk transmission of scrapie from sheep, even limited exposure to milk from goats can pose significant risk for scrapie transmission to both goat kids and lambs.

## Introduction

Classical scrapie is a naturally transmissible, fatal neurodegenerative disease of sheep and goats (small ruminants). Like other transmissible spongiform encephalopathies (TSEs), classical scrapie has a variable pre-clinical period (2–5 years) that culminates in neurologic signs which may include pruritus, gait changes, tremor, visual impairment, and changes in behavior [1, 2]. The transmissible agent is referred to as a ‘prion’ and a hallmark of infection is conversion of the normal cellular form of the prion protein into a misfolded, disease-associated form (PrP^Sc^) that has a proteinase K resistant core (PrP^res^) [3]. During the course of classical scrapie infection in small ruminants, PrP^Sc^ progressively accumulates in peripheral lymphoid tissues and the brain [4–6]. There is no treatment and the economic impact of scrapie to sheep and goat industries in the United States is estimated to be $10–20 million annually [7]. Classical scrapie is also a significant problem for small ruminant industries abroad. As an example, more than 10,500 cases have been diagnosed in goats from some member states of the European Union over a 15-year period [8]. Determining modes of transmission is a critical factor in mitigating the occurrence of new cases and in achieving and sustaining eradication of classical scrapie.

The risk of prion transmission in small ruminants is highest during the neonatal period. In sheep, a major source of neonatal exposure to prions is the placenta [9]. In addition, transmission can occur via colostrum and milk from scrapie-infected sheep [10, 11] and may be enhanced if the ewe (female sheep) is co-infected with small ruminant lentivirus (SRLV) [12, 13]. In comparison to sheep, the placenta of goats accumulates only sparse amounts of PrP^Sc^ [14] but is nonetheless highly infectious to lambs and goat kids (young sheep and goats, respectively) [15]. While transmission of scrapie prions to lambs via goat milk has been demonstrated, transmission was only detected in lambs subject to prolonged consumption of milk totaling at least 38 liters [16, 17]. The goal of the present study was to assess neonatal transmission of scrapie prions to both lambs and kids via limited exposure to milk from does (female goats) in the preclinical phase of disease.

## Materials and methods

### Ethics statement

All animals in this study were maintained under animal care and use protocols approved by the Washington State University Institutional Animal Care and Use Committee (Animal Subjects Approval Forms 03811, 03815, and 04107).

### Donor goats and milk collection

Milk donors consisted of two Nubian-Saanen crossbred goats (G4204 and G4205) that were born to a doe with naturally-acquired scrapie (the placenta donor in [15]). The donor goats were maintained in a small group of goats of similar age with unlimited access to shelter and water. Animals were fed a balanced ration of grass and alfalfa hay and provided free access to appropriate salt and mineral supplements. All goats were observed daily by animal care staff with additional observations conducted at least monthly by a veterinarian (DAS). Prion protein genotypes were determined by PCR amplification of the open reading frame of the prion protein gene (*PRNP*) and sequence analysis according to published methods using DNA isolated from EDTA-anticoagulated blood [18, 19]. Prior to milk collection, preclinical scrapie infection of the donors goats was confirmed by scrapie immunohistochemistry (IHC; details below) applied to rectal biopsy samples collected once (Table 1).

**Table 1:** Scrapie status and milk collection from donor goats.

	G4204	G4205
Age at positive rectal biopsy (days)*	658	621
Age at parturition (days)	774	740
Age at milk collection (days)	859–873	833–847
Total volume of milk collected (liters)	15.165	15.075
Age at first record of clinical signs (days)	943	943
Age at cull (days)	958	946

* Positive result as detected by immunohistochemistry for accumulation of PrP^Sc^ within rectoanal mucosa-associated lymphoid tissue with a cocktail of anti-prion monoclonal antibodies F99/97.6.1 and F89/160.1.5.

Milk collection occurred during weeks 13 and 14 of lactation after the does’ offspring were weaned. Collection was performed once or twice daily for about 2 weeks after which the mammary glands were dry-treated to facilitate healthy cessation of milk production. Somatic cell counts in the milk were determined within 30 minutes after collection using an automated cell counter (DeLaval cell counter DCC; DeLaval International AB) [20]. The milk was stored in 0.5 or 1 liter bottles at -80°C for approximately 20 months before use. The donor goats were closely monitored for clinical signs of scrapie and subsequently euthanized by venous administration of a commercially available barbiturate solution. Postmortem tissues were fixed in formalin and processed for assessment of PrP^Sc^ accumulation by scrapie IHC (described below) [21, 22].

### Recipient animals and milk feeding

Milk recipients selected for this study were born to goats or sheep with no known exposure to scrapie. The genotype of all milk recipients was determined by PCR amplification and sequence analysis of the open reading frame of *PRNP* as previously described [18, 19]. Saanen goat kids (n = 4) and mottled-faced lambs (n = 4; Suffolk X Rambouillet or Suffolk X Targhee) nursed colostrum from their dams for approximately 24 hours after which they were transported to isolation rooms for inoculation and group housing according to species. To minimize the risk of carryover transmission from previous studies, isolation rooms were thoroughly cleaned and then fogged using a 40% bleach solution for at least one hour. Using this procedure, carryover transmission has not been detected during other experiments in which inoculated sheep failed to acquire scrapie (n = 4 confirmed by mouse bioassay) and when sentinel animals were co-housed with inoculated animals [23, 24]. The number of milk recipients chosen per species reflects a sample size that minimizes animal use yet provides reasonable power to detect transmission events under an assumption of moderate risk. Specifically, the experimental design provides a power of 0.8 at a nominal significance level of α = 0.05 when the true proportion is 0.2 (i.e. transmission to one-in-four exposed animals).

Aliquots of milk inocula from G4204 and G4205 were transferred from -80°C to -20°C two to three weeks prior to use. All kids and lambs were individually offered the milk inoculum via a bottle five times per day. At the time of feeding, frozen milk from the specified donor was selected (irrespective of day within the collection period) and placed under warm tap water to thaw and warm. Any milk not consumed during a single feeding was stored at 4°C and similarly warmed and offered at the subsequent feeding. Additional milk was thawed as needed until the inoculation volume had been consumed. All inoculation volumes were consumed by either 72 or 96 hours of age. Following inoculation, kids were fed fresh cow’s milk and lambs were fed lamb artificial milk replacer until weaning. During and after weaning, goats and sheep were offered a balanced ration of grass and alfalfa hay and appropriate salt and mineral supplements. All animals received vitamin D_3_ (Vitamin A D injection, Agri Laboratories; 75,000 IU per milliliter dose) beginning at approximately 5 weeks of age and every 4 to 5 weeks thereafter due to being housed indoors for the duration of the study. Young animals received a 0.5 ml dose subcutaneously and were transitioned to a 1 ml dose at 4 to 5 months of age. At the time of cull, euthanasia was performed by administration of a commercially available barbiturate solution (pentobarbital) into the circulatory system. Postmortem tissues were collected and either fixed in formalin or frozen (-20°C or -80°C) for assessment of PrP^Sc^ or PrP^res^ accumulation, respectively.

### Immunohistochemistry

Antemortem biopsy samples of recto-anal mucosa associated lymphoid tissue (RAMALT) were obtained after application of topical analgesic (2% lidocaine hydrochloride gel) and positioning of a speculum [25]. Antemortem biopsies were collected from goat milk donors at 20–21 months of age and from milk recipients beginning at 12 months post inoculation (mpi) and repeated thereafter at six month intervals until cull. Tissues collected postmortem from milk donors and recipients included obex, tonsil, retropharyngeal lymph nodes, ileocecal junction, and ileocecal lymph nodes. Scrapie IHC was performed, as previously described [21, 22]. Briefly, 3 to 5 μm sections of formalin-fixed, paraffin-embedded tissues were labeled using a cocktail of monoclonal antibodies (F99/97.6.1 at 10 μg/ml and F89/160.1.5 at 10 μg/ml; cell culture supernatant from hybridomas grown in-house lot numbers 01272014A and 06022014A, respectively; both mouse IgG_1_ isotype) or with monoclonal antibody F99/97.6.1 alone. Primary antibody labeling was followed by labeling with a secondary antibody (DISCOVERY Universal Secondary Antibody; Ventana Medical Systems), reagents from a FastRed kit (DISCOVERY RedMap kit; Ventana Medical Systems) and counterstaining with hematoxylin. For antemortem RAMALT samples, a minimum of 10 follicles per section were assessed for PrP^Sc^ accumulation at each biopsy time point.

Mammary gland tissue and supramammary lymph nodes were also collected postmortem from goat milk donors G4204 and G4205. Sequential sections (4 μm) of formalin-fixed, paraffin-embedded mammary tissue and lymph nodes were prepared. One section was stained with hematoxylin and eosin (H&E) and the adjacent section was subjected to scrapie IHC using F99/97.6.1 (10 μg/ml) as described above.

### Immunoblot analysis

Accumulation of PrP^res^ in lymph nodes was assessed by immunoblot as previously described [14] with the following modifications. Homogenates of ileocecal lymph node or retropharyngeal lymph node were prepared at 15% (w/v) in lysis buffer consisting of 10 mM Tris-HCl pH 7.5, 0.5% NP-40, 0.5% sodium deoxycholate. For standard immunoblot analyses, homogenates were treated with collagenase (25 mg/ml for 2 hours at 37°C) prior to proteinase K treatment (400 μg/ml for 60 minutes at 37°C). Sample loading buffer (NuPAGE LDS Sample Buffer; Invitrogen) was added and samples were electrophoresed through 12% Bis-Tris protein gels (Invitrogen). Proteins were transferred to PVDF membrane for immunodetection with F99/97.6.1 (2.4 μg/ml) and HRP-conjugated goat anti-mouse IgG_1_ secondary antibody (1:5000; Southern Biotechnology). Bound antibody was detected by chemiluminescence (Amersham ECL) captured on film.

When PrP^res^ was not detected by the standard immunoblot protocol described above, samples were treated with sodium-phosphotungstic acid (Na-PTA) to enrich for PrP^res^ [26, 27]. Briefly, lymph node homogenates (15% w/v) were incubated overnight at 37°C with either 25 mg/ml collagenase or 0.05% trypsin-EDTA. Homogenates were then mixed 1:1 with 4% Sarkosyl and incubated at 37°C for 15 minutes prior to treatment with 100 μg/ml DNase at 37°C for 45 minutes. Samples were pelleted by centrifugation at ~1300 x g for 5 minutes and treated with proteinase K (200 mg/ml for 60 minutes at 37°C). Finally, Na-PTA (4% w/v in 170 mM MgCl_2_, pH 7.4) was added to a final concentration of 0.3% Na-PTA and samples were incubated at 37°C for 60 minutes. Enriched PrP^res^ was pelleted by centrifugation at 20,500 x g for 30 minutes, dissolved in water prior to the addition of sample loading buffer, electrophoresed, and transferred to PVDF. Immunodetection was performed with an antibody cocktail consisting of F99/97.6.1 (2.4 μg/ml) and P4 (0.1 μg/ml; r-Biopharm) followed by goat anti-mouse IgG (H+L, Fab) antibody conjugated to HRP (1:6000; KPL, Inc.) and chemiluminescence detection.

### Assessment of small ruminant lentivirus infection

The animals were also tested for small ruminant lentivirus (SRLV) infection. Blood samples were submitted from all goat milk donors, goat milk recipient kids and their does, and goat milk recipient lambs and their ewes to the Washington Animal Disease Diagnostic Laboratory (Pullman, WA) for detection of serum antibodies against SRLV. The competitive ELISA used for this assessment does not discriminate antibodies directed against caprine arthritis encephalitis virus (CAEV) and ovine progressive pneumonia virus (OPPV).

Donor goat milk leukocytes were assessed for CAEV provirus by a quantitative PCR (qPCR) assay. DNA was isolated from 2–3 million cells using the Puregene cell DNA purification kit (Qiagen) and quantified using a Nano-drop spectrophotometer. One microgram of DNA was tested in triplicate in a CAEV qPCR assay that used the same components and conditions as a previously described OPPV qPCR assay [28] with primers and probes specific to CAEV as described in [29]. A plasmid containing a portion of CAEV-63 *env* (Genbank accession M60855; 1904-2760bp) was developed to create a copy number reference standard. Template DNA was isolated from goat synovial membrane cells infected *in vitro* with ~10^6^ TCID_50_ of CAEV-63 over 2 weeks. Amplification of CAEV-63 *env* was performed using previously published primers [29] with PCR amplification conditions as follows: 1 cycle at 95°C for 4 min; 35 cycles of the following: 95°C for 30 sec, 50°C for 30 sec, and 72°C for 2 min; 1 cycle of 72°C for 7 min; 4°C indefinite. The 857bp *env* fragment from CAEV-63 was cloned into TOPO pcDNA2.1 (Invitrogen) and sequenced using M13 forward primer, M13 reverse primer, and dye terminator methods. The resulting CAEV-63 *env* plasmid was diluted to 10^7^ copies/μl and diluted 10-fold serial dilutions with 5 μg/ml herring sperm DNA in sterile water for standard curve generation.

## Results and discussion

**Fig 1:**
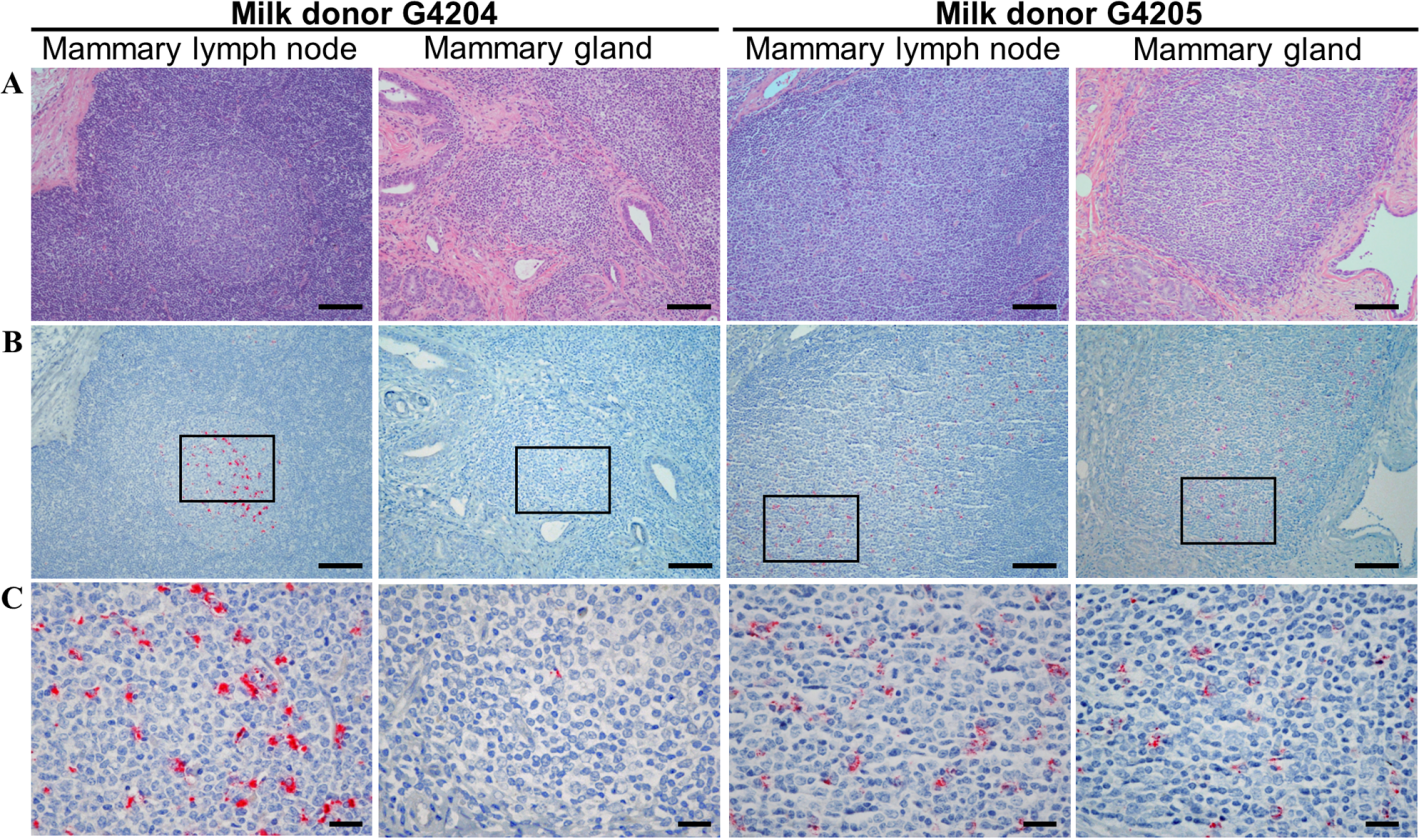
Lymphohistiocytic mastitis and PrP^Sc^ accumulation in milk donor goats co-infected with scrapie and SRLV. Supramammary lymph nodes and mammary gland tissues from milk donor goats G4204 (left 2 columns) and G4205 (right 2 columns) were collected postmortem, formalin-fixed and paraffin-embedded. Serial sections were either stained with hematoxylin and eosin (A) or subjected to scrapie immunohistochemistry (B, C). **(A)** Shown are follicles within supramammary lymph nodes (columns 1 and 3) and dense inflammatory infiltrates within mammary gland tissue (lymphohistiocytic mastitis; columns 2 and 4). **(B)** PrP^Sc^ accumulation (red chromogen) was located within germinal centers of the lymphoid follicles and within lymphohistiocytic infiltrates of the mammary glands. Overtly more PrP^Sc^ was observed in the mammary gland of G4205 as compared to G4204. Areas highlighted by boxes in (B) are shown at a higher magnification in **(C)**. Scale bars: 100 μm (A, B); 20 μm (C).

Previously, the presence of prions in milk from sheep with classical scrapie had been directly demonstrated by ewe-to-lamb transmission studies [10–13] and indirectly by the detection of prion protein misfolding activity [11, 30, 31]. Since much less was known about the presence of prions in the milk of infected goats, the goal of this study was to evaluate the potential for transmission of classical scrapie from goats to both goat kids and lambs by the oral route. At the time milk was collected for storage, the donor goats were positive for PrP^Sc^ accumulation in RAMALT but clinical signs associated with classical scrapie were not yet evident (Table 1). Clinical signs were first detected in the donor goats approximately 2 to 3 months after milk collection and does were euthanized by 31.5 months of age. Postmortem analyses confirmed the presence of PrP^Sc^ in the obex and peripheral lymph tissues of the donor goats (S1 Fig). Studies suggest that inflammation associated with SRLV infection enhance PrP^Sc^ accumulation in sheep and goats [32, 33]. Cell culture models have also shown an increased accumulation of PrP^Sc^ due to SRLV [34]. Thus, the milk donor goats were tested for SRLV at 20 months of age and found to be serum positive for antibody against the virus. Co-existence of naturally-acquired SRLV and scrapie in both donor goats was supported by observations of lymphohistiocytic mastitis, a pathology commonly associated with SRLV infection, and PrP^Sc^ accumulation in the mammary gland (Fig 1; see S2 Fig for comparison to mammary tissue from goats without SRLV). Accumulation of PrP^Sc^ was observed in 10 of 10 slides representing different regions of the mammary gland from G4205. Accumulation of PrP^Sc^ appeared less abundant per section of mammary gland from G4204 and was observed in only 6 of 10 slides. Further, SRLV provirus was detected by quantitative PCR [28, 29] in leukocytes isolated from the milk of each donor goat at approximately 14 weeks postpartum (i.e., a single sample obtained during the two week milk collection period). SRLV provirus was lower in G4204 (approximately 27 copies of CAEV per microgram DNA) when compared to G4205 (approximately 380 copies of CAEV per microgram DNA). Thus, similar to previous studies [12, 13], the mammary gland accumulation of PrP^Sc^ in goats appeared proportionally related to the mastitis and SRLV infection load. The combined observations of serology, lymphohistiocytic mastitis, and provirus in milk leukocytes further established that donor milk reserved for this study originated from goats with naturally-acquired classical scrapie and SRLV.

Numerous studies have demonstrated the impact of genetics on susceptibility to classical scrapie. In goats, wild type haplotypes encoding proline or serine at codon 240 (P_240_ or S_240_) of *PRNP* (haplotypes 1 and 2, respectively [19]; detailed in Table 2 footnote) are susceptible to classical scrapie whereas other haplotypes with polymorphisms encoding serine at codon 127 (S_127_), methionine at 142 (M_142_), serine at codon 146 (S_146_), histidine at 154 (H_154_), glutamine at 211 (Q_211_), or lysine at 222 (K_222_) are associated with extended incubation times or possible resistance [35–40]. The donor goats (G4204 and G4205) were heterozygous for *PRNP* haplotypes 1 and 2 (Table 2). Saanen goat kids selected for this study were also of scrapie susceptible genotypes (G4619, G4620, and G4621: heterozygous for haplotypes 1 and 2; G4625: homozygous for haplotype 1). The impact of donor and recipient *PRNP* genotype-matching on the efficiency of scrapie transmission as it relates to attack rate has been demonstrated in sheep [41]. Codon 240 in sheep is not known to be polymorphic and encodes serine [42, 43]. Thus we selected recipient lambs that were homozygous for a scrapie susceptible haplotype encoding alanine at codon 136, arginine at codon 154, and glutamine at codon 171 (ARQ/ARQ; Table 2) with no additional polymorphisms [44], i.e., matching *PRNP* haplotype 2 in goats. Shortly after sheep were inoculated for this study, it was shown that ARQ/ARQ sheep were more susceptible than VRQ/VRQ (valine at codon 136) sheep to scrapie when inoculated orally with goat brain tissue [16]. Thus, our selection of ARQ/ARQ sheep for inoculation should have provided the most senstive small ruminant model for detection of classical scrapie derived from goats.

**Table 2:** Milk donor and recipient relationships and *PRNP* genotypes.

Animal ID	Donor or Recipient	Dam ID	Sire ID	Genotype*
Goats				
G4204	Donor	G3950	G4061	1,2
G4205	Donor	G3950	G4061	1,2
G4619	Recipient	G4478	G4584	1,2
G4620	Recipient	G4478	G4584	1,2
G4621	Recipient	G4475	G4584	1,2
G4625	Recipient	G4187	G4584	1,1
Sheep				
S4631	Recipient	S4604	Sx9977	ARQ/ARQ
S4632	Recipient	S4604	Sx9977	ARQ/ARQ
S4633	Recipient	S4611	Sx9977	ARQ/ARQ
S4634	Recipient	S4611	Sx9977	ARQ/ARQ

* Two haplotypes are wild type in goats as described by White and colleagues [19]: haplotype 1 is G_127_I_142_H_143_N_146_ R_154_R_211_Q_222_P_240_ and haplotype 2 is G_127_I_142_H_143_N_146_ R_154_R_211_Q_222_S_240_. The ARQ haplotype in sheep is wild type [44] and includes 112_M_141_L_136_A_154_R_171_Q_.

**Fig 2:**
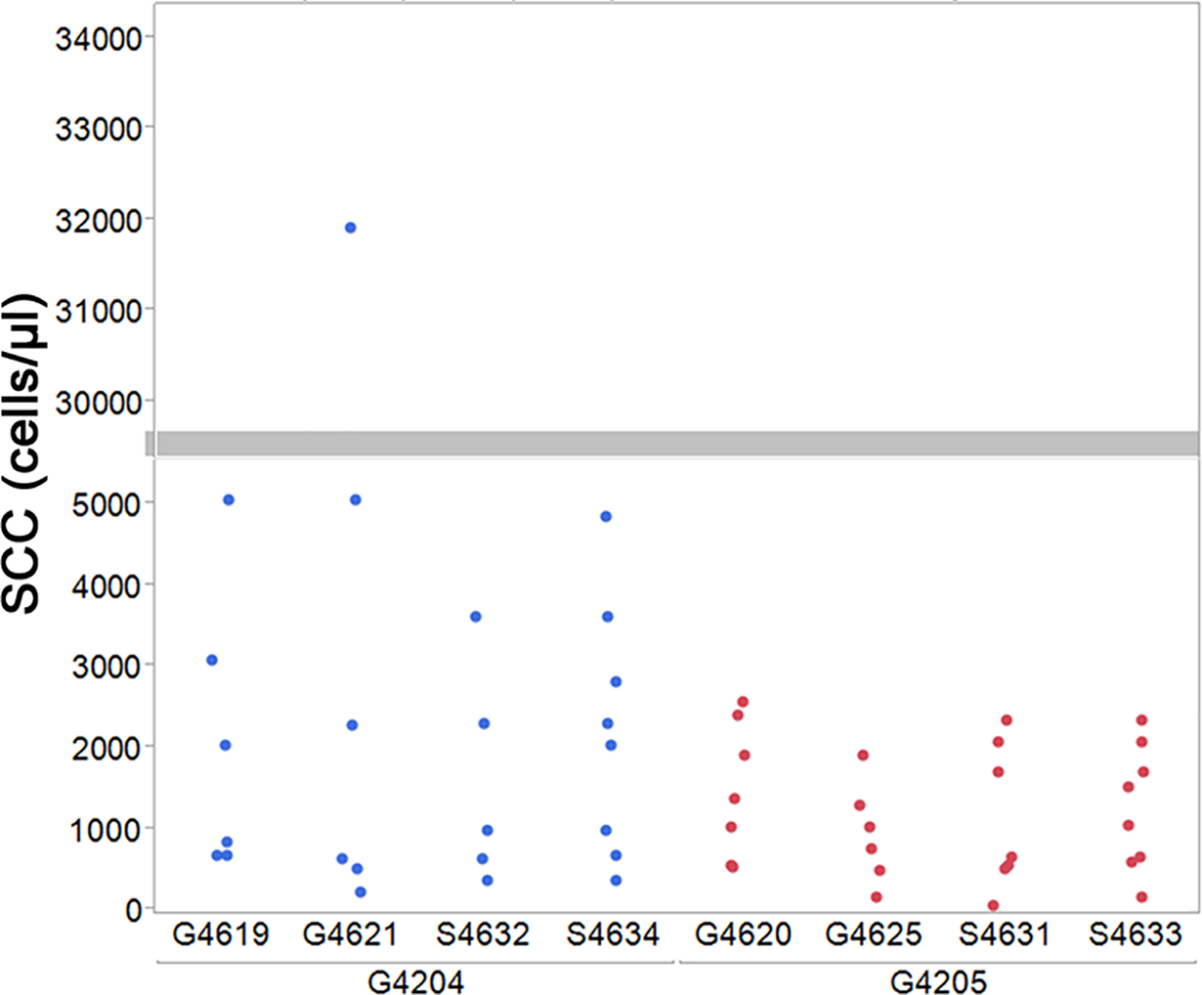
Somatic cell counts in goat milk inocula. Milk was collected during weeks 13 and 14 of lactation from two goats in the pre-clinical phase of scrapie infection. Somatic cell counts of the milk inocula are grouped first by milk donor (G4204, blue dots; G4205, red dots) and then by milk recipient (G46## = goat kids; S46## = lambs). Somatic cell count per microliter of milk as was determined using a DeLaval Cell Counter DCC. The grey bar denotes a break in the y-axis.

Milk from each donor goat was fed to two recipient goat kids and two recipient lambs. Goat kids were inoculated with 3.625 to 4.5 liters of milk; all lambs received 3.7 liters of goat milk. Inoculated milk volumes are approximately equivalent to one gallon of milk. Twin lambs or kids (Table 2) received milk from different donor goats. The median somatic cell count in milk from G4204 was 2024.5 cells/μl (minimum: 225 cells/μl; maximum 31,915 cells/μl) whereas milk from G4205 had a median somatic cell count of 1016.5 cells/μl (minimum: 53 cells/μl; maximum 2561 cells/μl) (Fig 2). There was no significant difference in the somatic cell counts of the milk used for inoculation but values were greater than what would typically be observed in milk from healthy goats (i.e. goats without SRLV) [45, 46]. Shortly after inoculation of animals for this study, a study from the United Kingdom reported transmission of scrapie via milk from goats to sheep [16, 17]. While our study confirms this finding in lambs and extends it to goat kids, it also demonstrates transmissibility in the context of a North American isolate of scrapie. Further, our study demonstrates transmission after relatively low volume ingestion of mid-lactation milk (i.e. no colostrum) from goats in the pre-clinical phase of scrapie. The volumes of milk inocula used in our study were 10-fold lower than that which transmitted scrapie and approximately half the lowest inoculated volume of milk used in the study by Konold and colleagues [16, 17].

Evidence of classical scrapie transmission via goat milk was first assessed by antemortem testing for PrP^Sc^ accumulation in all milk recipient sheep and goats. Biopsy samples of the RAMALT were assessed for the presence of PrP^Sc^ by scrapie IHC and observations are summarized in Table 3. PrP^Sc^ was detected in one sheep (S4632) at 18 mpi; two additional sheep (S4631 and S4633) had detectable PrP^Sc^ at 24 mpi. Accumulation of PrP^Sc^ in RAMALT was not observed in the biopsy samples from the remaining sheep or any of the goats. Observed transmission of classical scrapie from goat milk to sheep at 18 mpi was similar to previous observations even though sheep in the current study consumed approximately one-tenth the volume of goat milk that resulted in scrapie infection in a study by Konold and colleagues [16, 17, 47]. Animals in the current study were housed as groups (sheep separate from goats) and lateral transmission from ovine milk inoculated sheep to co-housed flock mates has been described previously [10]. Although lateral transmission between S4632 and S4631 or S4633 cannot be ruled out, evidence of infection was detected within a reasonably tight 6 month time frame among the positive animals. In addition, the lack of detectable PrP^Sc^ in S4634 further supports that positive antemortem tests in the other sheep resulted from consumption of the milk inoculum at birth and not lateral transmission from the sheep S4632 that was positive for PrP^Sc^ at 18 months post inoculation.

**Table 3:** Antemortem detection of SRLV and PrP^Sc^ in goat (G) and sheep (S) recipients of milk from goats co-infected with pre-clinical scrapie and SRLV.

					Rectal Biopsy Result^‡^			
Milk Donor	Volume inoculated	Milk Recipient	Sex*	SRLV status^†^	12 mpi	18 mpi	24 mpi	30 mpi
G4204	3.7 L	S4632	M/C	N	ND	P	P	NT
	3.7 L	S4634	M/C	N	ND	ND	ND	ND
G4205	3.7 L	S4631	F	N	ND	ND	P	NT
	3.7 L	S4633	F	N	ND	ND	P	NT
G4204	3.7 L	G4619	F	P	ND	ND	ND	ND
	4.5 L	G4621	M/C	P	ND	ND	ND	ND
G4205	3.7 L	G4620	M/C	N	ND	ND	ND	ND
	3.625 L	G4625	F	P	ND	ND	ND	ND

* M/C = castrated male. F = female.

^†^ SRLV = small ruminant lentivirus. P = positive, N = negative as determined by serology test.

^‡^ mpi = months post inoculation. P = positive result as detected by scrapie immunohistochemistry for PrP^Sc^ with the anti-prion monoclonal antibodies F99/97.6.1 and F89/160.1.5. ND = PrP^Sc^ not detected. NT = not tested.

Co-infection of scrapie and SRLV has been associated with increased peripheral distribution of PrP^Sc^, including increased accumulation in the mammary gland [32, 48]. There is also evidence suggesting SRLV (specifically maedi-visna virus) enhances transmission of scrapie via milk from sheep [12, 13]. The milk used for inoculation in the current study was collected from goats positive for both SRLV and scrapie. Since SRLV can be efficiently transmitted via milk [49], serologic testing to determine SRLV infection status in all milk recipients was conducted at 13–14 months of age to ensure any SRLV antibody passively absorbed as neonates would no longer be present in recipient blood (reviewed in [29]). Three of the four inoculated goat kids (G4619, G4621, and G4625) tested positive for antibody to SRLV despite being born to does that were negative for antibody to SRLV (Table 3). Although cross species transmission of SRLV has been documented [50], no antibody to SRLV was detected in the four recipient sheep despite nursing colostrum from their SRLV-positive dams and being fed SRLV-infected goat milk during the first 3 to 4 days of life. Whereas SRLV may have contributed to detectable PrP^Sc^ accumulation in the mammary gland of the milk donors in the current study, antemortem detection of PrP^Sc^ in the recipient sheep and goats appears to have been independent of co-transmission of SRLV.

**Fig 3:**
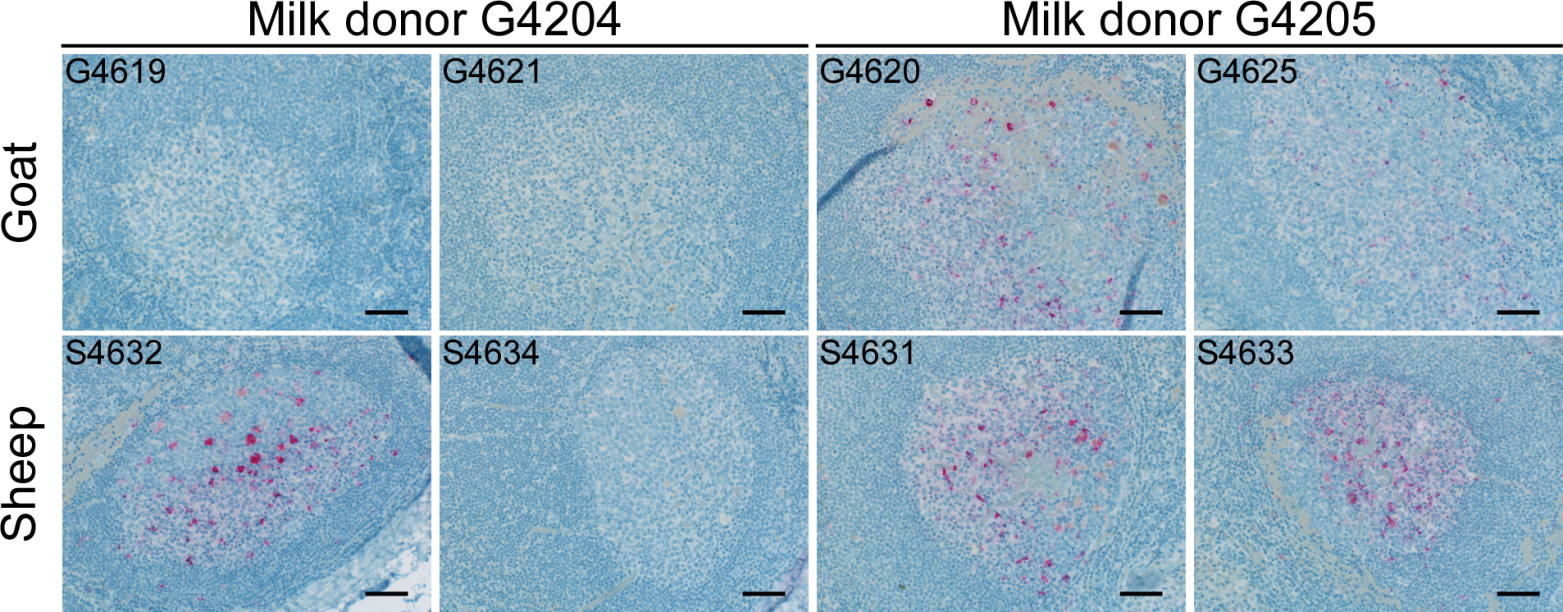
Postmortem PrP^Sc^ detection in goats and sheep inoculated with milk from goats with pre-clinical scrapie. Retropharyngeal lymph nodes were collected postmortem from goats (top row) or sheep (bottom row) inoculated with milk from donor G4204 (left two columns) or G4205 (right two columns). Evidence of scrapie transmission was detected in postmortem tissues collected at approximately 33 months post inoculation by scrapie immunohistochemistry in two of four goats inoculated as kids (top row) and three of four sheep inoculated as lambs (bottom row). Red chromogen deposits = PrP^Sc^ detected using a cocktail of anti-prion monoclonal antibodies F99/97.6.1 and F89/160.1.5; scale bar is 50μm.

Clinical signs of scrapie were not apparent in any of the recipients prior to euthanasia at approximately 33 mpi. Collection of tissues at cull was similar to what would be performed on apparently healthy sheep and goats that are subject to regulatory scrapie slaughter surveillance in the United States [51]. Scrapie IHC was applied to postmortem tissues to assess for the presence of PrP^Sc^. In sheep (Table 4), PrP^Sc^ was only detected in the postmortem tissues of the three recipients with positive rectal biopsies (i.e., S4631, S4632, and S4633). In these three sheep, PrP^Sc^ was observed in formalin fixed retropharyngeal lymph node (Fig 3) as well as other lymphoid tissues examined, and the dorsal motor nucleus of the vagus nerve. In addition, homogenates of frozen ileocecal and retropharyngeal lymph nodes were assessed by western blot as early accumulation of proteinase K resistant PrP (PrP^res^) is often first detected in these lymphoid tissues. In concordance with the scrapie IHC results, PrP^res^ was only detected in the three sheep with positive scrapie IHC findings. The similar tissue distribution and relative accumulation of PrP^Sc^/PrP^res^ in 3 of 4 sheep suggests transmission of scrapie occurred from neonatal ingestion of the milk inoculum. While lateral transmission cannot be ruled out, the lack of postmortem detection of PrP^Sc^/PrP^res^ in the fourth sheep further supports transmission from the original inoculum as this sheep had been housed with the group for the entire 33 months of the experiment with one of the cohorts testing positive 15 months prior to the end of the experiment.

**Table 4:** Postmortem detection of PrP^Sc^/PrP^res^ in goats and sheep inoculated with milk from pre-clinical, scrapie infected goats.

			Immunohistochemistry					Western blot	
Milk Donor	Milk Recipient	Days post inoculation	Ileocecal Junction	Ileocecal LN*	RPLN	Tonsil	Obex	Ileocecal LN	RPLN
G4204	S4632	975	P^†^	P	P	P	P	P	P^PTA^
	S4634	975	ND	ND	ND	ND	ND	ND	ND
G4205	S4631	975	P	P	P	P	P	P	P
	S4633	975	P	P	P	P	P	P	P^PTA^
G4204	G4619	990	ND	ND	ND	ND	ND	ND	ND
	G4621	987	ND	ND	ND	ND	ND	ND	ND
G4205	G4620	990	P	P	P	P	P	P^PTA^	P
	G4625	985	P	ND	P	P	P	ND	P

* LN = lymph node. RPLN = retropharyngeal lymph node

^†^ P = positive result as detected by scrapie immunohistochemistry for PrP^Sc^ with the anti-prion monoclonal antibodies F99/97.6.1 and F89/160.1.5 or western blot for PrP^res^ using F99/97.6.1 as the primary antibody. P^PTA^ = positive western blot result following sodium phosphotungstic acid enrichment of PrP^res^. ND = PrP^Sc^ or PrP^res^ not detected.

Sensitivity of antemortem RAMALT biopsy in goats can be poor in some situations [5, 52], thus a postmortem examination was conducted on all goat recipients at approximately 33 mpi, similar to the sheep recipients. Although none of the four recipient goats were positive by antemortem rectal biopsies, PrP^Sc^ was detected in postmortem tissues from two of the recipients (Table 4). PrP^Sc^ was detected in the retropharyngeal lymph nodes (Fig 3), other lymphoid tissues, and in the obex of goat recipients G4620 and G4625, both of which received milk from donor goat G4205. Western blot detection of PrP^res^ accumulation was concordant with scrapie IHC findings in these two goats. In contrast, evidence of scrapie infection could not be demonstrated by either method in the two goats fed milk from G4204. Since goat kid and lamb recipients received similar volumes of milk, the results of this bioassay suggest that prion titer in milk from donor goat G4204 (transmission to 1 of 4 recipients) may have been less than in milk from G4205 (4 of 4). The observed difference between the donor goats in accumulation of PrP^Sc^ in mammary gland tissue (Fig 1) supports the possibility of a prion titer difference in their milk but this is only a subjective measure as transmission via sheep milk has been observed in the absence of detectable PrP^Sc^ in the mammary gland [12]. While bioassay is a gold standard method for measuring infectious titer, prion protein misfolding activity—as measured by benchtop methods including protein misfolding cyclic amplification (PMCA), may serve as a surrogate measure of infectivity [53, 54]. Misfolding activity, an essential mechanism of prion replication (reviewed in [55, 56]), has been detected by PMCA in milk from sheep with scrapie [31] and was associated with scrapie infectivity [11]. In contrast, misfolding activity has proven difficult to be reliably detected in the milk of scrapie infected goats, having been observed in only one of three replicates from 2 of 56 milk samples collected from 14 infected goats [17]. Several aliquots of milk from the current study were clarified according to the methods of Maddison and colleagues [31] and subjected to 5 rounds of serial PMCA consisting of 48 cycles of sonication and incubation [57]. Brain homogenate prepared from transgenic mice expressing ovine *PRNP* (tg338) provided the normal cellular prion protein substrate in all reactions. In an effort to increase the efficiency of misfolding activity in the PMCA assay, some experiments included polyadenylic acid [17, 58] or zirconia/silica beads [59] in the reaction tubes during cycling. Despite our own experience using bioassay and PMCA assay to study scrapie [23, 57], we have not been successful in detecting misfolding activity in aliquots of the goat milk used in this study. Even though the scrapie prion titer in milk from G4204 is uncertain and may have been low relative to milk from G4205, it was nonetheless sufficient to transmit infection by low volume feeding to at least one lamb (S4632).

The design of this study was such that the number of sheep and goats receiving milk inoculum would allow for a reasonable chance to detect transmission should at least moderate prion infectivity be present in milk from goats with scrapie. Although transmission was observed in three of four sheep and two of four goats, this minimal design does not provide sufficient statistical power to determine if sheep were more susceptible than goats to these goat milk inocula. As was indicated above, all inoculated goats and sheep were of scrapie susceptible genotypes with no polymorphism known to delay incubation of scrapie. All sheep were paternal half-siblings, as were all goats. Thus, genetic susceptibility was suspected to be similar within each species and does not explain the lack of transmission to some of the recipient goats and sheep. Aside from the potential differences in prion titer between our sources of milk inocula, it is possible that the way in which milk was selected for feeding may have contributed to the observed variation in transmission. In most cases, milk collected on different days during weeks 13 and 14 of lactation were fed to different animals. Our results suggests that prion shedding may be inconsistent from day to day but do not indicate if more shedding occurred during one week over the other. Another possibility is that the infectivity found in milk is associated with milk leukocytes. While the somatic cell count tended to be higher in milk from G4204 than G4205 (Fig 2), there was no significant difference in the median somatic cell count whether grouped by donor or recipient for analysis. The inoculum used for S4634, G4619, and G4621 contained the highest median somatic cell counts yet these animals demonstrated no evidence of scrapie transmission as was assessed by IHC and immunoblot. Thus, a clear cause of the variation in transmission observed between the two species remains to be elucidated.

## Conclusions

The observations described herein demonstrate scrapie transmission via milk from goat to goat kid, confirm cross-species transmission to sheep, and demonstrate that consumption of approximately 4 liters of milk (about a gallon) from pre-clinically affected goats can transmit scrapie to both goat kids and lambs.

Mention of trade names or commercial products in this article is solely for the purpose of providing specific information and does not imply recommendation or endorsement by the US Department of Agriculture.

## Supporting information

S1 Fig Accumulation of scrapie-associated prion protein (PrP^Sc^) in the obex hindbrain and retropharyngeal lymph node of the milk-donor goats. Accumulation of PrP^Sc^ (red chromogen) was similar in both goats (G4204 shown in A, B and C; G4205 in D, E and F). Accumulation was variable but widespread throughout the obex hindbrain (A and D). Advanced accumulation of PrP^Sc^ in the dorsal motor nucleus of the vagus nerve was accompanied by spongiform degeneration (B and E: magnifications of regions outlined in A and D). Accumulation of PrP^Sc^ was also present in follicles of the retropharyngeal lymph node (C and F). Tissues counterstained with hematoxylin. Scale bars: A and D = 2 mm; B and E = 50 μm; C and F = 100 μm.
(TIF)

S2 Fig Supramammary lymph node and mammary gland reference images from goats without SRLV infection. **(A)** Hemotoxylin and eosin staining of supramammary lymph node and mammary gland from an SRLV-negative and scrapie-negative goat (G4202, left 2 columns) and from an SRLV-negative but clinical scrapie-positive goat (G4330, right 2 columns). **(B)** and **(C)** Detection of PrP^Sc^ accumulation by scrapie immunohistochemistry. Mammary gland inflammation and PrP^Sc^ accumulation in supramammary lymph nodes and mammary glands were not observed in goat G4202. Accumulation of PrP^Sc^ was observed in supramammary lymph nodes of G4330 but neither PrP^Sc^ accumulation nor inflammation were observed in the doe’s mammary glands. Boxes in (B) highlight areas of higher magnification shown in (C). Red chromogen deposits = PrP^Sc^ detected using anti-prion monoclonal antibodies F99/97.6.1; (A) and (B) scale bar is 100 μm, (C) scale bare is 20 μm.
(TIF)
