# Supplementary figures and images for: Low-volume goat milk transmission of classical scrapie to lambs and goat kids
Source: PLoS One. 2018 Sep 20;13(9):e0204281. doi: 10.1371/journal.pone.0204281 (PMC6147516; doi:10.1371/journal.pone.0204281)

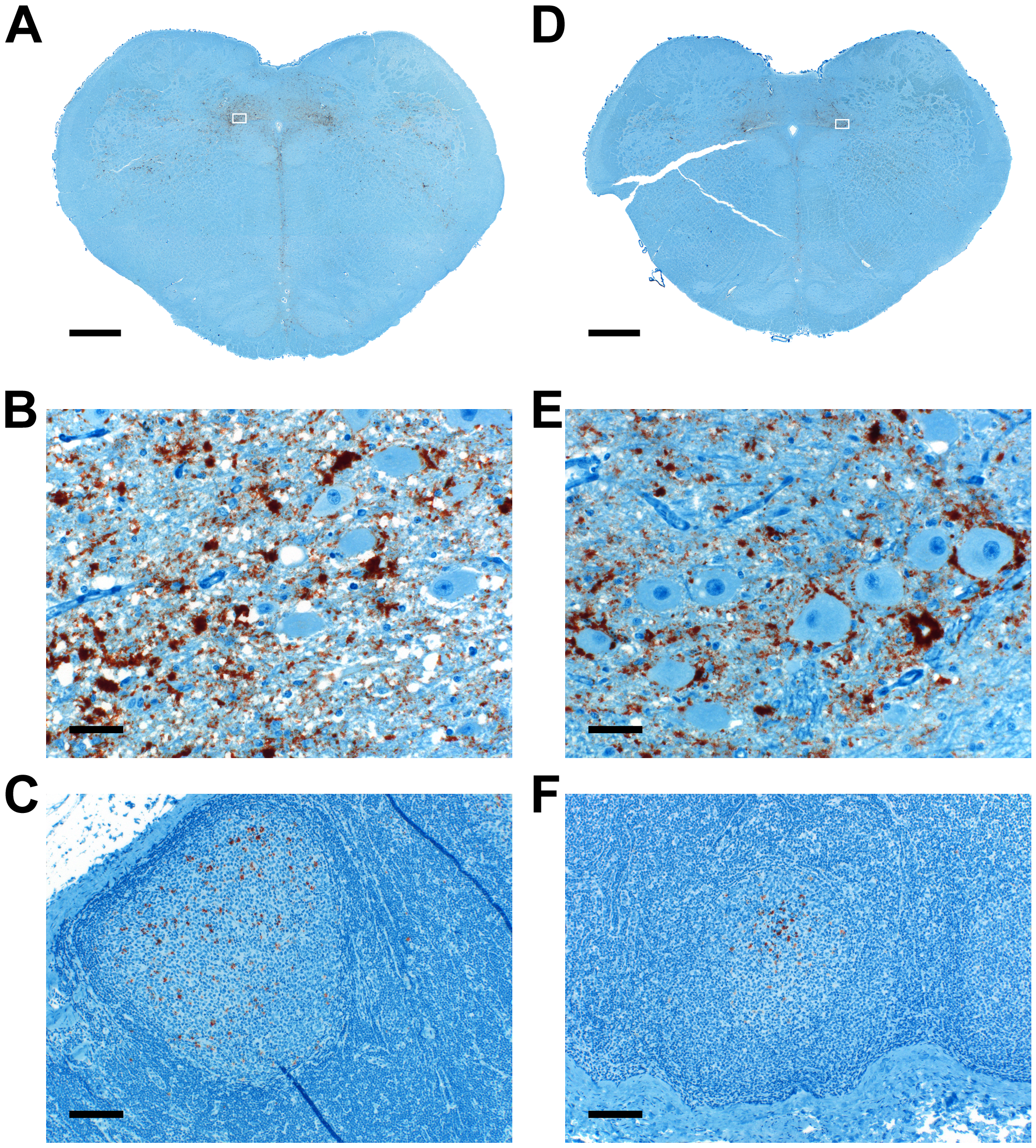

Supplement: S1 Fig — Accumulation of PrPSc (red chromogen) was similar in both goats (G4204 shown in A, B and C; G4205 in D, E and F). Accumulation was variable but widespread throughout the obex hindbrain (A and D). Advanced accumulation of PrPSc in the dorsal motor nucleus of the vagus nerve was accompanied by spongiform degeneration (B and E: magnifications of regions outlined in A and D). Accumulation of PrPSc was also present in follicles of the retropharyngeal lymph node (C and F). Tissues counterstained with hematoxylin. Scale bars: A and D = 2 mm; B and E = 50 μm; C and F = 100 μm. (TIF) [file pone.0204281.s001.tif]

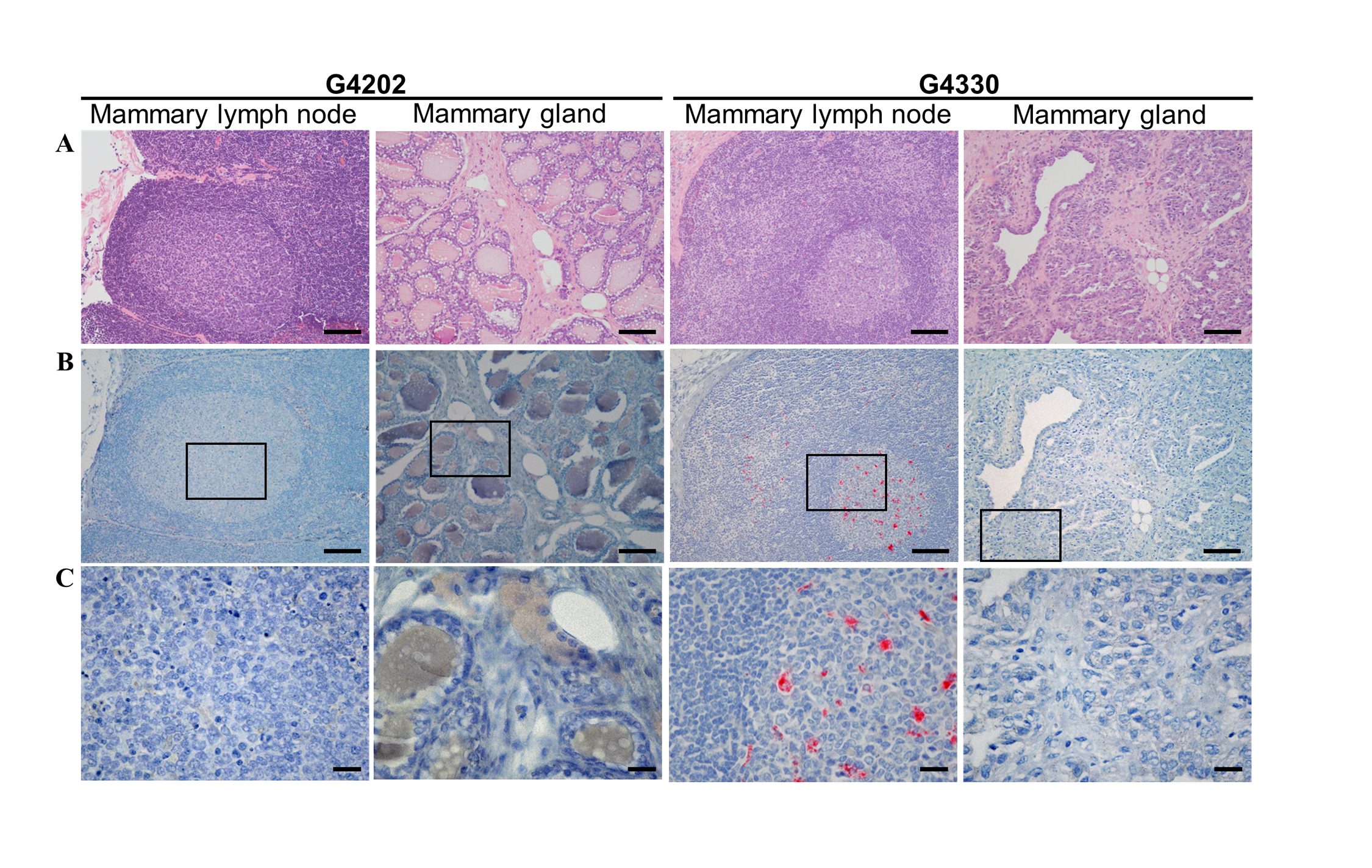

Supplement: S2 Fig — (A) Hemotoxylin and eosin staining of supramammary lymph node and mammary gland from an SRLV-negative and scrapie-negative goat (G4202, left 2 columns) and from an SRLV-negative but clinical scrapie-positive goat (G4330, right 2 columns). (B) and (C) Detection of PrPSc accumulation by scrapie immunohistochemistry. Mammary gland inflammation and PrPSc accumulation in supramammary lymph nodes and mammary glands were not observed in goat G4202. Accumulation of PrPSc was observed in supramammary lymph nodes of G4330 but neither PrPSc accumulation nor inflammation were observed in the doe’s mammary glands. Boxes in (B) highlight areas of higher magnification shown in (C). Red chromogen deposits = PrPSc detected using anti-prion monoclonal antibodies F99/97.6.1; (A) and (B) scale bar is 100 μm, (C) scale bare is 20 μm. (TIF) [file pone.0204281.s002.tif]
